# Validation and Reliability of an Assessment Tool for Tracheal Intubation Skills Among Anesthesiology Residents: A Pilot Study

**DOI:** 10.7759/cureus.89716

**Published:** 2025-08-10

**Authors:** Yuko Akanuma, Mari Sakuma, Seiki Abe

**Affiliations:** 1 Nursing, International University of Health and Welfare, Narita, JPN; 2 Anesthesiology, St. Luke's International Hospital, Tokyo, JPN; 3 Anesthesiology, St. Luke’s International Hospital, Tokyo, JPN

**Keywords:** advanced practice nurses, airway management, perianesthesia nursing, simulation education, task shift

## Abstract

Background: Although junior residents at this hospital are required to receive anesthesiology training, there are no standards for evaluating tracheal intubation skills, and the training is currently at the discretion of each instructor. Therefore, as peri-anesthesia nurses, we have been involved in airway management education and collaborated with the anesthesiology department to develop skills standards. The purpose of this study was to improve airway management education and the quality of medical safety by examining the reliability and validity of the assessment chart.

Methods: This was a prospective observational study in which peri-anesthesia nurses and anesthesiologists developed an assessment chart to measure tracheal intubation skills. The study was conducted at a simulation center from August to September 2021, with five volunteer junior residents. After the first day of training, a series of actions from tracheal intubation to ventilator connection were video recorded on a simulator and scored by an expert physician using an evaluation chart and analyzed for reliability and validity.

Results: Reliability confirmed internal consistency (Cronbach's α=0.8), and intra-class correlation coefficients (ICCs) had strong correlations within the mean measures (ICC (2, 3) =0.78 (CI: 0.12-0.97)), indicating inter-rater reliability. Validity was compared with the standard score of 52 out of 100, defined for residents, and internal validity was confirmed, as all measures had strong correlations (r=0.9). In contrast, correlations between trauma avoidance items were poor (r=0.35, ns).

Conclusions: Validation of the reliability of the skills assessment chart revealed that it measured the competency of tracheal intubation of junior residents. For the items that had poor correlations, instruction by senior physicians and peri-anesthesia nurses, in collaboration, was strengthened to attain the standard. The plan was to create an educational environment in which residents, as members of the healthcare team, contribute to patient quality of life and medical safety.

## Introduction

Anesthesiology training is mandatory in the junior residency program at our hospital, St. Luke’s International Hospital, Tokyo, Japan, and anesthesiology has the advantage of providing experience in advanced technical skills, such as tracheal intubation, spinal anesthesia, and central venous catheter insertion. Anesthesiology is also a good opportunity to learn medical safety, prevention of medication errors and overdoses, working with multidisciplinary staff to learn non-technical skills, and learning the basics of safety management [1,2].

However, because the training period is short (one to two months), the number of experiences is likely to vary. In addition, the ability to acquire skills depends on the discretion of each instructor. It is difficult to define a single standard for proficiency in tracheal intubation because it varies greatly depending on the airway information and surgical procedure, and there are no uniform standards in Japan.

Indicators for assessing the acquisition of tracheal intubation skills include the educational guidelines of the Japanese Society of Anesthesiologists (Basic Course in Airway Management) [3] and the Anesthesia Education Standards Form (Milestones) [4] of the Accreditation Council for Medical Education of the United States. However, since neither is sufficiently specific for immediate clinical work, we created an evaluation chart that is consistent with the educational level and work environment of our hospital.

In this preliminary study, video recording of tracheal intubation maneuvers was performed on volunteer anesthesiology residents and scored based on visual information. The evaluation chart was examined for reliability and validity to determine if it adequately reflects improvement in tracheal intubation skills.

The purpose of this study was to develop and test the reliability and validity of a skill assessment chart for simulator-assisted tracheal intubation for junior anesthesiology residents. The significance of this study is that the standardization of tracheal intubation skills will enable objective evaluation. It will also improve the teaching level of senior physicians and increase awareness of the importance of education. Furthermore, the acquisition of tracheal intubation skills by trainee physicians will contribute to saving lives and improving medical safety in future clinical practice.

This study was presented and awarded the Best Presentation at the 46^th^ Annual Meeting of the Japanese Association for Operative Medicine in 2024.

## Materials and methods

The study design is a pilot study of a forward-looking observational study. The participants were five volunteer junior resident physicians. The study was conducted at our simulation center at St. Luke's International Hospital, Tokyo, Japan, from August to September 2021.

The tracheal intubation skills assessment chart was referenced because a value of Adam, R [5] was similar to the educational status in our anesthesiology department, and the questions were created independently, accounting for the environment of the anesthesiology department. Five goals were set for each stage of airway management, and questions were developed to achieve each goal. Questions were scored on a scale of one to five, with five points per question, with 20 questions, for a total maximum score of 100 points. To ensure that the content of the evaluation could be used objectively, the anesthesiologists and peri-anesthesia nurses discussed the appropriateness of the questions and agreed to a score of 52 as the score that our junior residents should achieve (Table 1).

**Table 1:** Endotracheal intubation skill evaluation form Each item is scored on a five-point scale, with a total of 20 items for a maximum of 100 points. Decimal scores are also acceptable. Scoring criteria are as follows: one point: unable to perform independently; critical errors such as esophageal intubation (beginner level); two points: able to perform with physical assistance; three points: able to perform with verbal guidance (general competency level); four points: able to perform independently; five points: performs skillfully (specialist level)

Goal	Description	Score
Goal 1	To be able to assume a position suitable for tracheal intubation	Score
1	Position the patient's head appropriately (sniffing position, inserting pillows for head and shoulders, etc.)	/5
Goal 2	To be able to properly insert a laryngoscope	
2	Holding the laryngoscope correctly	
3	Cross-finger opening	
4	Insertion from the right corner of the mouth, the tongue is moved to the left and reaches the midsection.	
5	Recognizes the epiglottis valley and can insert the blade	
6	Appropriate application of force to the epiglottis valley	
	Subtotal	/25
Goal 3	Obtain an appropriate airway field of view	Score
7	Blade position is appropriate, neither deep nor shallow	
8	Blade is in the midsection.	
9	Lift the epiglottis without tongue protrusion	
10	The initial laryngeal deployment angle is appropriate (about 45 degrees)	
11	Vocal cords are visible.	
12	Number of laryngeal deployments (Note: If you can do it once, you get 5 points.)	
	Subtotal	/30
Goal 4	To be able to correctly insert an intubation tube into the trachea and start artificial respiration	Score
13	No contact with other tissues during tube insertion	
14	Insert the tube to the proper depth	
15	Inject cuff and confirm placement in trachea (chest elevated, etCO2, auscultation, cuff leak)	
16	Initiate manual or mechanical ventilation	
17	Proper skin fixation with tape	
18	Number of times the tube is inserted (Note: If you can do it once, you get 5 points)	
	Subtotal	/30
Goal 5	To avoid trauma during intubation	Score
19	Not applying excessive force during laryngoscopy or tube insertion	
20	Laryngoscope does not pinch lips or cause dental damage	
	Subtotal	/10
	Overall score	point
	The time required	seconds

Data collection was explained by the peri-anesthesia nurse and consent was obtained from the participants prior to orientation on the first day of training. This was followed by an airway management orientation using the Laerdal Airway Management Simulator (Laerdal Medical AS, Stavanger, Norway). Specifically, the course of action included airway mechanics, mask ventilation, tracheal intubation, and a series of actions to confirm tracheal intubation and connection to a ventilator.

**Figure 1:**
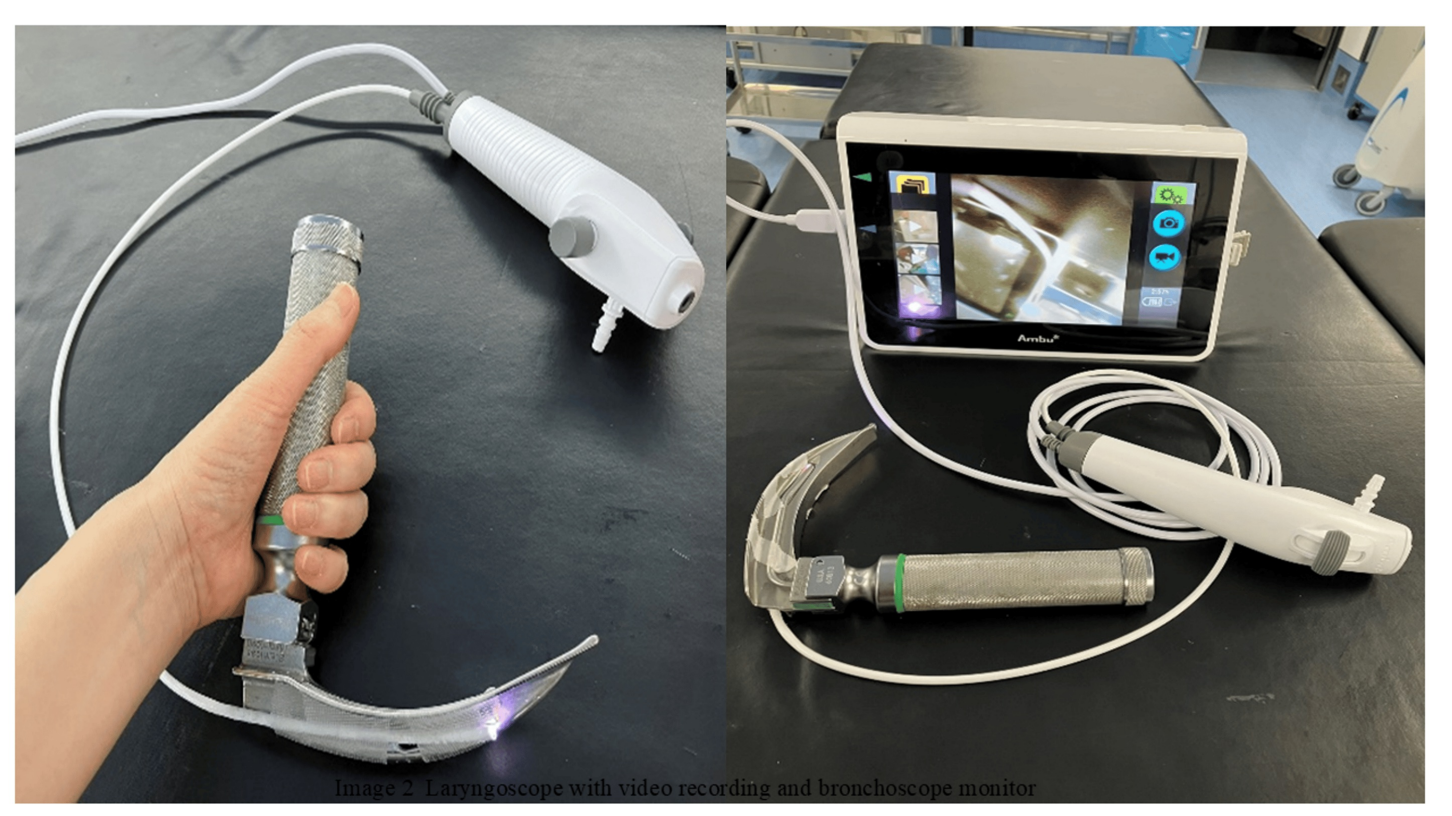
Laryngoscope with video recording and bronchoscope monitor

After several repetitions of practice, a video was recorded from two directions, the oral cavity and the perspective of the resident, as a confirmation test of this sequence of actions for the acquisition of the research data. A Macintosh laryngoscope with a disposable bronchoscope glued to it and an Ambu® bronchoscope monitor (Ambu A/S, Ballerup, Denmark). The view from the perspective of the resident was recorded using a smartphone. Finally, we ensured that the faces of the residents were not captured in the images, taking care to prevent individual identification. The video recordings were subsequently scored by a third-party anesthesiologist using an evaluation chart (Figure 1).

For the analysis of the evaluation scores, Cronbach's α coefficient and the intra-class correlation coefficient (ICC) were calculated for verification of reliability. For verification of validity, the correlation coefficients between the gold standard score (52) and the scores that the specialist calculated, and the relationship between each goal and the overall score, were also determined. This study was approved by the research ethics review committee of St. Luke's International Hospital, Tokyo, Japan (approval number: 21-R087).

## Results

The tracheal intubation skills of five resident volunteers were video recorded and scored by the specialist on the evaluation chart. First, for subject characteristics, there were five male and no female subjects. By grade, two were first-year residents and three were second-year residents. The second-year residents were new to anesthesia. In each specialty area, four residents were in internal medicine and one in surgery.

Cronbach's α coefficient was as high as 0.8 for internal consistency, as a test of reliability. Furthermore, the ICC was also high at 0.78 (95% CI: 0.12-0.97, N=5), confirming the inter-rater reliability. For validation, the gold standard was defined as a total score of 52 when specialist physicians scored the video, and the mean score of the five participants ranged from 46.6 to 60 points. Compared to the gold standard, there was a high correlation coefficient of 0.9, indicating that the specialist physicians were able to score the videos appropriately (Table 2).

**Table 2:** Evaluation of the assessment tool N=5; p<0.05; Reliability analysis and intra-class correlation analysis were performed using IBM SPSS Statistics software, version 29 (IBM Corp., Armonk, NY).

Reliability	Index	p-value​	f-value​	95%CI​
Cronbach's alpha coefficient	0.81			
Intra-class correlation coefficient	0.78	p<0.05​	5.36	0.12~0.97​
Validity				
Correlation coefficient (participant total score vs. gold standard)	0.99			

Next, correlation coefficients were calculated to determine the impact of each goal on the total score. There was a significant correlation coefficient of 0.9-1.0 (p<0.05) for Goal 2 (proper laryngoscope insertion) to Goal 4 (correct tracheal tube insertion and initiation of mechanical ventilation). However, Goal 1 (proper patient positioning for tracheal intubation) and Goal 5 (avoid injury during intubation) were lower and less statistically significant than the other goals (r=0.35-0.87, ns). During the video recording, the residents were primarily focused on the manipulation of the oral tube and were not aware of cervical retroflexion and airway mechanics. This phenomenon showed that the residents were not aware that they were causing tooth damage and lip lacerations (Table 3).

**Table 3:** Effect of each learning goal on the total score N=5; p<0.05; p<0.001, *Not significant; Spearman‘s correlation coefficient using Microsoft Excel (Microsoft Corp., Redmond, WA)

Goal	Goal description	r	p-value​
Goal 1	Proper patient positioning for tracheal intubation​	0.87​	ns*​
Goal 2	Proper laryngoscope insertion​	1.00​	p<0.001​
Goal 3	Optimal proper laryngeal view​	0.90​	p<0.05​
Goal 4	Correct tracheal tube insertion and initiation of mechanical ventilation​	0.94​	p<0.05​
Goal 5	Avoid injury during intubation (excessive force, lip laceration, dental injury)​	0.35​	ns*​

## Discussion

The reliability and validity of the tracheal intubation skills assessment chart were determined. Anesthesiologists and peri-anesthesia nurses involved in education at our hospital collaborated to determine the questions used in the assessment chart, and they formulated five objectives and 20 questions. This section discusses the analytical process and results from the formulation of the questions to the implementation of the evaluation. The significance of residents, specialists, and nurses working together to promote education and research as part of a medical safety team will be discussed.

Reliability of the evaluation table

Reliability was calculated from the scores of the three specialists and the five residents, and the ratings were comparable. The analysis shows that Cronbach's alpha coefficient was as high as 0.8, which confirms the internal consistency of the ratings. Other authors have reported a reliability coefficient of 0.8 for the kappa coefficient [6] and a Cronbach's alpha of 0.97 for the retest method with a small sample of 10 persons [7], which is comparable to the results of the present study. In addition, there was a high correlation of 0.78 for the ICC, which shows the agreement, stability, and accuracy of the ratings, as ICC incorporates intra-rater and inter-rater measures.

Previous studies have used the direct observation of procedural skills (DOPS) score [8,9], but all were scored by only selecting "yes/no" or by using a five-step scale. There are minimal studies in which the DOPS score was calculated as a whole number or analyzed using an interval scale, as in the present study. Therefore, this study proposes a new method for analysis at a higher scale level. In addition, the retest method and the establishment of a target group may be considered as possibilities for future studies.

Validity of question items for assessing skills

The evaluation chart was based on our specific criteria, in which specialists and peri-anesthesia nurses collaborated to confirm the internal validity. Of importance in this preliminary study was whether the rating chart accurately measured tracheal intubation skills and whether the validity of the content could be confirmed as a reproducibility index. This was discussed with statistical experts and the specialists, and a reasonable standard score for the residents was determined to be 52 points. As a result, the scores of the five participants scored by the three specialists had a very high correlation with the standard score, confirming the validity of the content of the questions.

Although ensuring reliability and validity is fundamental in scale development, there are some studies that have only reported reliability [7, 10]. However, since the validity of the content of the questions is a factor in determining whether a skill is being accurately measured, confirmation of the validity of the content of the questions was a favorable outcome. In addition, criterion-related validity is how the measure relates to external criteria. DOPS [11,12] is a widely used assessment instrument in education and practice that is not limited to tracheal intubation skills, and the assessment uses observation of a scenario in which the procedure is performed. The objective structured assessment of technical skills (OSATS) [7, 13] educational index is often used in medical education, and it uses simulators and simulated patients. DOPS and OSATS are both scored on a five-point scale for each item and are analyzed on nominal and ordinal scales. The present evaluation chart is a new method in which all 20 items are analyzed as a total score integer, and it has potential in the development of new standards.

The correlation coefficients for each goal that affected the overall score were measured. Goal 2 (proper laryngoscope insertion), Goal 3 (optimal proper laryngeal view), and Goal 4 (correct tracheal tube insertion and initiation of mechanical ventilation), which measure tracheal intubation skills, had very high correlation coefficients. Therefore, it was determined that the structure of the content was appropriate. In contrast, Goal 1 (proper patient positioning for tracheal intubation) and Goal 5 (avoid injury during intubation) were not significantly correlated and did not contribute to the total. During the video recording, the residents were focused on the manipulation of the intubation tube in the oral cavity and were unable to maintain an appropriate position to observe cervical retroflexion and airway mechanics.

In addition, they failed to consider tooth protection and lip laceration, resulting in excessive pressure being applied to the patient. Although this phenomenon is often observed at the junior resident level, appropriate safety management is required as a member of the medical team. Peri-anesthesia nurses should be responsible for physician orientation to enhance education that considers patient care, including awareness of medical safety and quality of life perspectives [14,15] to avoid undue positioning and trauma.

Expanded role of peri-anesthesia nurses in the medical team and education

As peri-anesthesia nurses, we believe that airway management should be more than placing a tube into the trachea or having a high success rate. Airway management should encompass positioning during intubation, appropriate mask ventilation, confirmation of intubation, and smooth transition to a ventilator. Patient care requires careful attention to the prevention of unnecessary trauma and reduction of adverse events, such as tooth damage and pharyngeal hemorrhage. Even in the short initial training period, the trainee should also be taught medical safety and human quality of life perspectives [14, 15] as a member of a medical team; therefore, it is a requirement that appropriate positioning and avoidance of trauma are achieved.

To the best of our knowledge, we were the first peri-anesthesia nurses in Japan with approximately 10 years of clinical practice, and as nurses in the Department of Anesthesiology, we have collaborated with physicians in a number of anesthesiology services, from pre-operative outpatient care to surgical anesthesia and postoperative management [16]. The decision to assume responsibility for resident airway management education was the result of this proven track record and trust. The resident rotation is very short (one to two months), and since anesthesiology is the only area in which tracheal intubation is systematically taught, our responsibility was significant. The results of this study confirmed that peri-anesthesia nurses can lead initial education and research, and it has contributed to the improvement of their status. We wish to continue to contribute to the expansion of the role of peri-anesthesia nurses by leading patient care and medical safety initiatives in collaboration with physicians as members of the medical team.

Limitations of the study

Although the evaluation chart was aligned with the clinical training and faculty level at our institution, the results are comparable to those of other well-established evaluation criteria. However, this pilot study was conducted at a single institution with a limited number of anesthesiology residents, which may constrain the generalizability of the findings. The brief duration of training may have influenced skill acquisition, and the low correlation coefficients observed for Goal 1 (patient positioning) and Goal 5 (injury prevention) suggest potential concerns regarding the construct validity of these specific items. Furthermore, the assessment tool was not benchmarked against established external evaluation frameworks such as OSATS or DOPS, limiting its external validity. Future research involving larger sample sizes and longitudinal designs is warranted to further substantiate the reliability and validity of this evaluation instrument.

## Conclusions

As peri-anesthesia nurses, we have developed criteria and conducted a preliminary study of the tracheal intubation skills of junior anesthesiology residents. The results of the study demonstrated the reliability and validity of the assessment chart and confirmed that the study could proceed.

For future studies, increasing the sample size to improve the accuracy of reliability and re-examination of the evaluation chart in comparison with other standards, with regard to validity, are required. For head positioning in Goal 1 and avoidance of trauma in Goal 5, for which the correlations were poor, discussion with senior physicians to reach standard scores resulted in an agreement that the educational perspective of care, with consideration for patient quality of life, required strengthening. As peri-anesthesia nurses, we will continue to lead the medical team and provide educational instruction to improve patient safety and care.
